# Ten-Year Clinical and Functional Outcomes of Anterograde Calcaneo-Stop Arthroereisis for Idiopathic Flexible Flatfoot in Children: A Single-Center Cohort Study

**DOI:** 10.3390/children12081047

**Published:** 2025-08-09

**Authors:** Giovanni Trisolino, Marco Ramella, Valeria Pizzuti, Marco Todisco, Stefania Claudia Parisi, Tosca Cerasoli, Gino Rocca

**Affiliations:** Pediatrics Orthopedics and Traumatology, IRCCS Istituto Ortopedico Rizzoli, 40136 Bologna, Italy; giovanni.trisolino@ior.it (G.T.);

**Keywords:** idiopathic flexible flatfoot, calcaneo-stop, subtalar arthroereisis, C-Stop, C-Stop results, Tegner Activity Scale, Foot and Ankle Ability Measure

## Abstract

Background: Idiopathic painful flexible flatfoot (FFF) in childhood can persist into adulthood, yet long-term data on subtalar arthroereisis via the calcaneo-stop (C-Stop) procedure are scarce. We aimed to evaluate clinical and functional outcomes at ≥10 years post-surgery and compare them with age-matched normative values in healthy populations. Methods: We conducted a single-time-point long-term follow-up on a subset of 232 children (age 10–14 years) selected from a retrospective cohort of 494 patients who underwent bilateral anterograde C-Stop between 2010 and 2014. Inclusion required idiopathic symptomatic FFF refractory to conservative care and a minimum 10-year follow-up. At a mean follow-up of 12.1 ± 2.5 years, patients completed the Foot and Ankle Ability Measure (FAAM) and Tegner Activity Scale (TAS). Secondary data included anthropometrics, implant details, accessory procedures, screw removal, and complications. Results: Respondents demonstrated excellent function: FAAM total 98.8 ± 3.7 (range 75–100) with 87.5% achieving the ceiling score; FAAM–ADL 99.3 ± 3.2; FAAM–Sport 98.0 ± 6.4. The mean TAS was 3.7 ± 2.0, with 53% active in sports—72% low-impact, 12% high-impact non-competitive, and 4% competitive. Sex and history of complications produced statistically significant but clinically small differences (<3% on FAAM total; <6 points on subscales). No outcome differences were observed by age or BMI, accessory procedures, or screw removal status. Conclusions: Ten years after C-Stop arthroereisis in childhood, patients exhibit functional scores comparable to normative values, high rates of ceiling effect on FAAM, and a modest level of physical activity predominantly in low-impact sports.

## 1. Introduction

Flatfoot is common in young children and is generally considered physiological. Prevalence is nearly universal under age 2, affects 45–70% of preschoolers (ages 2–5), and declines to 15–20% by age 10 [1,2,3]. In adults, it stabilizes around 15%, with slightly higher rates in males and individuals with obesity. As prevalence decreases with age and stabilizes around 10 years, many authors consider flatfoot beyond this age likely permanent, although the available evidence is largely based on cross-sectional studies [1,2,3]. Over half of these cases are flexible asymptomatic flatfeet, which are merely postural and aesthetic anomalies, not pathological conditions. Less than 10% of children have pathological or rigid flatfoot (linked to tarsal coalitions or neuromuscular conditions) or painful flexible flatfoot (FFF), often associated with obesity or generalized hyperlaxity. Idiopathic painful FFF, though infrequent, greatly concerns parents who frequently seek corrective treatments. Persistent symptoms, like pain, fatigue, cramping and related problems including excessive shoe wear and calluses, warrant treatment. Currently, there is no consensus on treating painful FFF; the prevailing recommendation is conservative treatment based on targeted exercises (stretching the gastro-soleus complex, strengthening the posterior tibialis, and/or insole support) reserving surgery for recalcitrant cases. Various surgical options exist, but there is no agreement on the optimal technique [1].

Surgical interventions for flatfoot, including soft-tissue procedures, arthroereisis, and osteotomies, yield diverse outcomes. Among these, subtalar arthroereisis (SA) has gained popularity, especially in Europe, due to its minimally invasive, joint-sparing nature [2] SA aims to limit excessive eversion and pronation of the subtalar joint, thereby improving talocalcaneonavicular alignment. The “calcaneo-stop” (C-Stop) procedure, a variant of SA introduced by Recaredo-Álvarez in 1970 and refined by Pisani [3,4], involves manually correcting talocalcaneal alignment using a screw partially inserted into the calcaneus (retrograde) or into the talus (anterograde) [5]. This screw secures the hindfoot in proper alignment and limits pronation through both mechanical and proprioceptive mechanisms. Unlike SA with endorthesis, the C-Stop screw is placed extra-articularly between the lateral tubercle of the talus and the anterolateral part of the calcaneus, avoiding the richly innervated sinus tarsi.

The C-Stop procedure has been increasingly adopted in pediatric orthopedic practice and is considered a promising surgical option for flexible flatfoot, particularly when conservative measures fail [1].

The benefits of this procedure include its minimally invasive and extra-articular nature, allowing for correction of hindfoot posture and alignment without altering bone structure or permanently restricting joint movement. Additionally, the procedure is reversible (the screw can be removed if intolerable) and does not significantly affect the possibility of future surgical interventions if needed. However, the efficacy of the C-Stop procedure remains debated. In particular, there is limited evidence regarding the long-term durability of favorable outcomes. Very few studies have reported results with minimum follow-ups longer than 10 years, when the child has become an adult and might experience a recurrence of symptoms and discomfort [6,7,8]. There is also a concern that patients may restrict their activities (especially sports) due to fears related to having undergone surgery, potentially leading to lower performance and more symptoms compared to the general population who do not have foot problems and have never had surgery.

Our study evaluated the long-term outcomes of the C-stop procedure for idiopathic FFF correction in childhood. We asked how young adults who underwent this surgery during childhood compare to age-matched normative values in clinical outcomes, functional performance, and physical activity. We hypothesized that ten years post-surgery, their clinical and functional scores would align with those of the general population.

## 2. Materials and Methods

### 2.1. Study Design

This study is a single-time-point long-term follow-up of a retrospective series of patients who underwent the C-Stop procedure for idiopathic symptomatic FFF during childhood at a single pediatric orthopedic surgery department. Our department is a tertiary referral center highly specialized for pediatric orthopedic surgery, performing approximately 18,000–20,000 outpatient visits and 1800–2000 pediatric surgical procedures annually. The study was conducted in accordance with the Declaration of Helsinki and approved by the Independent Ethical Committee “Comitato Etico di Area Vasta Emilia Centro” (32/2020/Oss/IOR) on 6 February 2020.

The hospital database was queried using specific codes to identify foot and ankle surgical procedures. From the identified cases, patients were selected based on specific inclusion and exclusion criteria. We included patients with symptomatic bilateral idiopathic FFF (symptoms persisting for over 6 months despite non-operative measures), graded 3 or 4 according to the Viladot Classification (based on plantar impression), with a rearfoot valgus > 10°, who underwent the bilateral C-Stop procedure between the ages of 10 and 14 from 2010 to 2014, with a minimum of 10 years of follow-up. This age range was selected because spontaneous correction is still expected under age 10, while after age 14, skeletal maturity necessitates alternative surgical strategies [9,10,11,12,13,14,15,16,17]. Furthermore, over 95% of our patients who undergo this procedure fall within this age range. We excluded patients with comorbidities such as cerebral palsy or genetic disorders, congenital abnormalities like longitudinal limb deficiency or tarsal coalition, mental impairments such as autism, or those who had additional lower limb surgeries for other anomalies like knee malalignment or hip problems. Also excluded were patients with conditions that could impact foot and ankle function, such as fractures, major trauma, tumors, bone and joint infections, or vascular accidents, as well as those with major deformities reported in their charts, unavailable charts, or incomplete questionnaire data. Baseline variables extracted from charts and radiographs included sex, age at surgery, weight, height, and age- and sex-adjusted BMI at the time of surgery. Additionally, data on any additional soft-tissue procedures like Achilles lengthening or tibialis posterior plication, screw removal (whether it was performed or not), age at screw removal, and pre- and post-operative radiographic variables such as calcaneal pitch were recorded. Complications, recurrence, and reoperations were also documented.

The hospital registry search identified 2189 cases treated between 2010 and 2014. Of these, 1063 were excluded due to discrepancies in diagnosis or treatment codes, and 635 were excluded based on eligibility criteria. This left 494 patients for contact.

### 2.2. Surgical Technique

**Figure 1:**
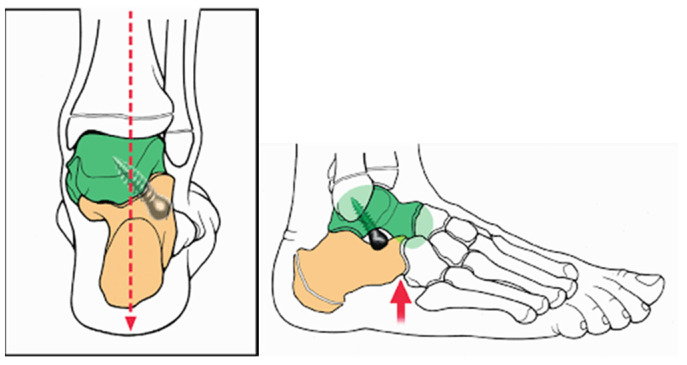
Schematic illustration of Spherus screw trajectory: inserted at a 45° lateral-to-medial angle in the coronal plane relative to the hindfoot axis (dashed red arrow indicating neutral calcaneal alignment), and a 0–35° posterior inclination in the sagittal plane (thick red arrow showing sagittal plane direction and arch elevation with restored joint alignment).

The anterograde calcaneo-stop procedure was performed under local anesthesia with sedation and a tourniquet. The patient was supine with the affected extremity slightly internally rotated and the foot in maximal supination and neutral dorsiflexion. A 1–2 cm incision was made at the sinus tarsi. The lateral tubercle of the talus was located and prepared for screw insertion using a straight awl, directed perpendicularly towards the medial malleolus. Under fluoroscopy, a specially designed screw (Spherus, Bioimpianti) was inserted at a 45° angle from lateral to medial on the coronal plane and 0 to 35° posteriorly on the sagittal plane (as depicted in Figure 1 [18]).

**Figure 2:**
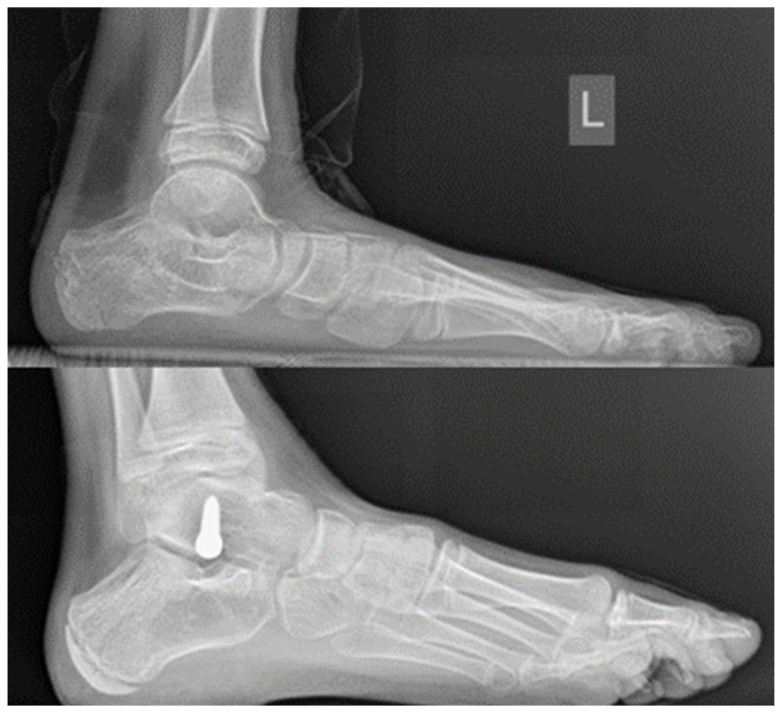
**Top**: pre-operative lateral radiograph demonstrating idiopathic flatfoot deformity. **Bottom**: intraoperative lateral radiograph confirming correct trajectory and final placement of the Spherus screw in the lateral talar tubercle.

This screw features a broad, tapered head with smooth, rounded edges, ensuring extensive contact with the sinus tarsi, distributing pressure and minimizing the risk of loosening or penetration. Crafted from titanium alloy, it provides exceptional biocompatibility and strength. The surgical technique has remained unchanged throughout the study period. From 2010 to 2016, the Spherus screw (Bioimpianti) was used, featuring a spherical head designed to improve load distribution and reduce the risk of calcaneal penetration compared to standard AO 4.5 mm screws or conical-head alternatives. Since 2017, a new model called Still screw (Dial Ortho) has been adopted, introducing a bottle-neck head design and a double-thread pitch to enhance talar fixation and reduce early loosening. Among the first 6415 implants with the Still screw, only 3 cases of breakage or cut-out were reported (0.05%). The screw head projected anterolaterally, effectively preventing excessive pronation while avoiding joint penetration. Heel alignment was assessed in dorsiflexion with the knee extended. Screw positioning is checked intraoperatively with a C-arm fluoroscopic shot (Figure 2).

If dorsiflexion was below −10°, triple-hemisection percutaneous Achilles tendon lengthening was performed using Hoke’s technique. If heel valgus persisted due to medial laxity, medial re-tensioning was achieved by plicating the posterior tibial tendon. Simple excision of the accessory navicular was performed if it was present and reported as painful during the preoperative examination.

Post-operatively, a walker brace was used for isolated C-Stop procedures and an ankle cast for combined soft-tissue procedures, each for three weeks. This was followed by an 8-week rehabilitation program with active foot mobilization, toe and heel walking, proprioceptive exercises, swimming, and cycling, avoiding other sports. After three months, regular sports, including competitive and contact activities, were gradually resumed. Screw removal was scheduled 24–26 months after the procedure, if requested, usually due to mild scar pain, screw discomfort, or slight joint restriction.

### 2.3. Outcome Variables

At a minimum of 10 years post-surgery, patients were recontacted. They were initially asked about any major conditions (e.g., fractures, significant trauma) that might have significantly affected their foot’s condition and function. Those without such issues were then asked to complete two questionnaires: the Foot and Ankle Ability Measure (FAAM) and the Tegner Activity Scale (TAS). Current weight, height, and type of sports activity were also collected.

The FAAM, derived from the Foot and Ankle Disability Index, consists of 29 items across two subscales: (1) Activities of Daily Living (FAAM-ADL): 21 items assessing difficulty with lower limb functions affecting ADLs (work, recreation); (2) Sport (FAAM-Sport): 8 items assessing difficulty with more complex sports-related activities. Each item is rated on a 5-point Likert scale (0–4), where 0 indicates complete inability and 4 means no difficulty. Scores for each subscale are calculated separately: FAAM-ADL ranges from 0 to 84, and FAAM-Sport from 0 to 32. The sum of the answered items is divided by the maximum possible score (84 for FAAM-ADL) and converted to a score out of 100, with higher scores indicating better function. FAAM has excellent reliability (test–retest ICC: 0.89–0.87) and validity (Cronbach’s alpha = 0.96; criterion validity r = 0.66), with no significant floor or ceiling effects [19]. The Minimal Detectable Change (MDC) is 5.7 points for ADL and 12.3 points for sport, while the Minimal Clinically Important Difference (MCID) is 8 points for ADL and 9 points for sport. The FAAM-ADL questionnaire was recently translated and validated in Italian [20], with normative data established for the European population (93.3% ± 11.6% for FAAM-ADL and 88.3% ± 16.3% for FAAM-Sport in individuals under 25) [21].

The TAS is a single-item questionnaire rated on an 11-point scale (0 to 10) according to the patient’s reported activity/work level, where 0 indicates maximum disability and 10 represents elite athletic performance [22]. It is a validated tool, cross-culturally adapted into Italian, with normative values reported for the general adult population (average 6.0 for men, and 5.4 for women) [23].

### 2.4. Sample Size Estimation

This study aimed to determine if patients who underwent the C-Stop procedure over 10 years ago had significantly different clinical and functional outcomes compared to normative data from healthy, age-matched populations reported in the literature. Sample size calculation was performed using the T-Test Means Difference from Constant function on G*Power (version 3.1) software. Our null hypothesis assumed no significant difference between our surgically treated cohort—patients operated on in childhood for idiopathic flexible flatfoot—and normative data from young, healthy athletes. According to the literature, such athletes, with no history of trauma or injury, typically score between 99.8% and 100% on the FAAM-ADL subscale [24,25]. To achieve 80% power for detecting a small effect size of 0.2, we used two-sided testing with a significant level of 5%, which required a sample size of 199 participants. Given the approximately 50% response rate observed in our previous long-term follow-up studies, we aimed to contact at least 400 patients to account for potential non-responses and loss to follow-up.

### 2.5. Statistical Analysis

Continuous variables are reported as mean ± standard deviation. Data distribution was assessed for normality, and the one-sample Wilcoxon signed-rank test was used to compare cohort means (FAAM-ADL and FAAM-Sport) with published normative values [21,25]. Between-group comparisons (e.g., by sex, complication status, accessory procedures, screw removal) were carried out using independent-samples *t*-tests or the Mann–Whitney U test, as appropriate, while categorical outcomes (complication rate, sports participation) were evaluated with chi-square tests. Associations among continuous measures (FAAM scores, TAS, age at surgery, BMI) were examined using Pearson correlation coefficients or Spearman’s rho when normality assumptions were marginal. A two-tailed *p*-value < 0.05 was considered statistically significant.

## 3. Results

Among the 494 eligible patients identified through the registry, 247 were either unreachable or did not complete the questionnaire, and 15 reported major conditions unrelated to the index surgery that could affect outcomes. Ultimately, 232 patients (47% response rate), were included in the final analysis, exceeding the initially required sample size by over 55%. We found no statistically significant differences between responders and non-responders in terms of demographics or surgical details (see Table S1 in the Supplementary Materials).

Baseline characteristics are reported in Table 1. The age at surgery was 11.5 ± 1 year; 4.9% of patients were obese; 7.9% of patients received additional procedures. Complications and recurrence were observed in 27 patients (5.5%. 95% CI: 3.5–7.5%) and were significantly more frequent among responders (8.2%) than non-responders (3.1%; *p* = 0.02). 

The mean age at the time of questionnaire administration was 23.8 ± 1.5 years, with a mean BMI of 22.3 ± 4.2 kg/m^2^. Among the respondents, 9.9% reported being overweight and 1.3% reported obesity.

The results are summarized in Table 2. FAAM-ADL scores were slightly but significantly lower than normative data for young athletes (mean difference: −0.5 points; 99% CI: −0.9 to −0.01; *p* < 0.01), while they were markedly higher compared to normative values from the general adult age-matched population (mean difference: 6.0 points; 99% CI: 5.7 to 6.4; *p* < 0.01). Overall, 87.5% of patients reported the maximum possible FAAM-ADL total score, with only 2.2% scoring below 90%.

Significant differences were observed between males and females, as well as between patients who reported complications or recurrences at the time of surgery and those who did not (see Tables S2 and S3 in Supplementary Materials). However, these differences averaged less than 8 percentage points, which corresponds to the MCID. Other variables such as age and BMI at surgery and follow-up did not significantly influence the outcome. No differences were observed between patients who underwent ancillary procedures and those who did not. Similarly, no differences were found between patients who had the screw removed and those who retained it, as the screw was not perceived as bothersome or problematic (see Supplementary Materials Table S3).

The TAS averaged 3.7 ± 2.0 points, showing a significant mean difference of 1 point between males and females. There was little to no correlation between the TAS and the FAAM-ADL, FAAM-Sport, age, weight, height, or BMI at follow-up. Among surveyed patients, only 53% participated in sports or work activities. Of these, 72% engaged in low-impact sports, 12% in high-impact sports (mostly non-competitive), and just 4% in competitive high-impact sports. Notably, there were no elite athletes in major leagues within this cohort.

In total, 85.5% of patients had the screws removed after a mean period of 2.3 ± 0.6 years. A subgroup analysis was conducted to compare outcomes between patients who underwent screw removal and those who retained the implant at final follow-up. No statistically significant differences were found in FAAM total, FAAM-ADL, FAAM-Sport, Tegner score, or complication rates between the two groups (Table 3).

## 4. Discussion

This study presents one of the largest single-center long-term follow-ups of pediatric patients treated with calcaneo-stop using a talar screw. Our findings are consistent with the current literature [1,26] and reinforce the safety and effectiveness of the procedure in treating idiopathic painful FFF, achieving functional outcomes comparable to, or better than, those reported in healthy young adults [8,26,27]. The surgical principles, established in the mid-1970s [4,5], have remained largely unchanged, reflecting the technique’s effectiveness and strong inter-operator reproducibility.

The mean FAAM-ADL (99.3 ± 3.2) and FAAM-Sport (98.0 ± 6.4) scores, confirm both the procedure’s efficacy and the long-term stability of hindfoot realignment, even after screw removal, surpassing published normative FAAM-ADL (93.3 ± 11.6) and FAAM-Sport (88.3 ± 16.3) values in young adults [21]. Despite these excellent results, only 53% of patients remained active in sports, predominantly low-impact disciplines (72%), with only 4% involved in competitive high-impact activities. Interestingly, Silva et al. reported similarly high short-term success (94% of 336 pediatric patients achieved a painless, corrected foot) and documented a rapid return to extracurricular sports, from 55% pre-removal to 77% by a mean follow-up of 41.3 ± 6.7 months after screw removal [28]. Although overall activity levels did not exceed baseline in their series, this early recovery highlights that mechanical correction reliably restores function in the mid-term; the persistence of only modest long-term sports engagement thus points more toward psychological and social factors, such as motivation, fear of injury, or changing interests, than to any residual biomechanical deficit. This apparent discrepancy of slightly lower FAAM-ADL scores compared to healthy young athletes [21,25], but clearly higher than age-matched normative values from the general population [24], likely reflects the specific characteristics of our cohort. While these patients report excellent functional outcomes, their relatively modest engagement in high-impact sports may explain why they do not fully match the athletic reference group. On the other hand, the lasting corrective effect of the calcaneo-stop procedure may result in better foot function compared to peers in the general population, where undiagnosed or untreated postural abnormalities can lead to symptoms in early adulthood.

Our study aligns with the current literature in reporting a 5.5% complication rate. These included 13 recurrences (only 1 of which required revision with a Grice procedure), 10 transient peroneal muscle spasms that resolved after screw removal, 3 cases of screw malposition requiring repositioning, and 1 superficial wound infection managed non-operatively. Notably, the majority of complications were minor and self-limiting. However, the higher rate of reported complications and failures among responders is noteworthy, as it highlights two distinct categories of issues: early peri-operative complications (e.g., wounds or screw-related problems, often transient and resolved after implant removal) and late complications (e.g., recurrence or arthritis-like pain). While these late issues were obviously more frequently reported by responders, they may also be present—if not more prevalent—among non-responders, suggesting that the true rate of unsuccessful outcomes may be underestimated in our study. Notably, patients who experienced complications or failures reported significantly lower functional outcomes than those without issues—a finding that, while of limited clinical relevance in our series, warrants further investigation. A recent systematic review analyzed 20 studies involving 1415 pediatric patients (2394 feet) who underwent the calcaneo-stop procedure. The authors reported significant improvements, including pain reduction in 93.5% of cases, improved heel alignment in 95%, high satisfaction in 95% of patients, and a low complication rate of 7.8% [1]. De Pellegrin and Moharamzadeh’s meta-analysis similarly documented low adverse-event rates (~8%) and high rates of arch restoration across subtalar arthroereisis techniques [26]. Moreover, Calvo Calvo et al.’s ten-year follow-up of the stop-screw technique demonstrated sustained radiographic correction and minimal recurrence after screw removal, mirroring our finding that elective removal, performed in 85.5% of patients at a mean of 2.3 ± 0.6 years, did not compromise long-term function as originally suggested by Recaredo-Álvarez [8]. However, it remains unclear whether removal is truly necessary, especially with modern, highly biocompatible titanium implants. Long-term outcomes appear comparable between patients who undergo screw removal and those who do not, provided there is no pain, dysfunction, or contracture.

Thus, we conclude that performing the intervention shortly before skeletal maturity, after age 10, when spontaneous correction is unlikely, allows patients to regain nearly perfect function, except for rare complications or recurrences. Several population-based studies confirm that the prevalence of pes planus decreases progressively with age, stabilizing between 15% and 35% in late adolescence and adulthood, and tends to be slightly higher in males [29,30,31,32]. These data support the concept that flatfoot persisting beyond age 10 is unlikely to resolve spontaneously, reinforcing the rationale for surgical correction before skeletal maturity. The long-term maintenance of correction after screw removal likely reflects structural and neuromuscular adaptations occurring during this key growth period, rather than continued dependence on the implant itself [1,6,8,33,34,35]. Current evidence suggests that temporary implant placement during a critical growth window induces neuromuscular and structural adaptations that persist even after screw removal. Although the causes of flexible flatfoot are multifactorial, including genetic, anatomical, and environmental factors, such growth-related remodeling may explain the long-term stability observed post-removal [2,10].

### Limitations

Despite these encouraging findings, the lack of an internal control group of untreated flatfeet or asymptomatic peers limits our ability to determine whether surgical correction yields superior outcomes to natural history or optimized conservative care. Sampling and recall biases may also exaggerate perceived gains, since treated patients, recruited from a specialized center, may report idealized current function and overestimate past limitations. Moreover, although we used published normative values from age-matched healthy cohorts as a reference, this indirect comparison has methodological limitations. Differences in population characteristics and data collection may affect comparability [36,37]. Moreover, the statistical method used (one-sample Student’s *t*-test) treats the reference value as fixed and does not account for its standard deviation or uncertainty. This can lead to highly significant *p*-values even for clinically negligible differences, as seen in our case. However, this approach is commonly accepted in pediatric orthopedic research when internal controls are not available [38]. Our focus on idiopathic FFF also precludes extrapolation to rigid or secondary deformities, which may require adjunctive procedures for optimal results [27].

Subtalar arthroereisis remains controversial given that many FFF are asymptomatic, raising questions about whether reported pain, fatigue, or activity avoidance truly reflect biomechanical deficits or parental concerns. Indeed, recent work indicates that even “silent” flatfeet are associated with lower quality-of-life scores across multiple domains and that parents often perceive discomfort more acutely than their children [39,40]. Such findings underscore the ethical imperative to weigh radiographic and clinical indications against each child’s subjective experience, motivation, and psychosocial context when considering C-Stop intervention.

Our retrospective design and reliance on e-mail surveys are further limitations: without pre-operative FAAM or TAS data, the magnitude of individual improvement remains imprecise, and the absence of repeat clinical exams or radiographs at final follow-up may miss subclinical relapse. Prospective, randomized studies comparing C-Stop to optimized conservative management, and incorporating baseline and serial PROMs, standardized imaging, and qualitative assessments of patient expectations, are needed to refine indications and ensure that surgical intervention delivers true, lasting benefits beyond the natural evolution of pediatric flatfoot.

## 5. Conclusions

The calcaneo-stop procedure is a minimally invasive surgical technique widely used to treat flexible flatfoot in pediatric patients. This approach has demonstrated significant efficacy in correcting foot deformity, alleviating symptoms, and improving quality of life. Key advantages of calcaneo-stop include a low complication rate, short recovery time, and the ability to avoid more invasive corrective surgeries. This study demonstrates a low risk of complications and recurrence, along with excellent and sustained clinical and functional outcomes, even following screw removal. Despite these advantages, the widespread adoption of C-Stop remains cautious: many FFF cases are asymptomatic, and many children with FFF who have never undergone surgery and have not shown any symptoms in adulthood, so the decision to operate should rest not only on radiographic severity but also on genuine symptom burden, patient motivation, and psychosocial context. Furthermore, children who experience difficulty participating in sports due to foot discomfort may not necessarily have a physical limitation but could instead be unmotivated, disengaged from the sport, or facing social challenges within the team or environment. Our findings underscore the importance of individualized assessment, as some children with untreated flatfoot maintain satisfactory function into adulthood. To refine surgical indications and avoid unnecessary interventions, future prospective studies should compare C-Stop with optimized conservative management, incorporate pre- and post-operative patient-reported outcomes, and explore the psychosocial determinants of activity resumption.

## Figures and Tables

**Table 1:** Baseline characteristics of 494 pediatric patients undergoing anterograde calcaneo-stop surgery, including demographic data, anthropometrics, implant details, accessory procedures, and complication rates.

Baseline Variables	Value
Patients (M/F)	494 (308/186)
Age at surgery (years)	11.5 ± 1.0 (10–14)
Weight (kg)	45.6 ± 7.6 (25–82)
Height (cm)	151.1 ± 6.9 (130–178)
BMI (kg/m^2^)	19.9 ± 2.4 (12.9–28.7)
Obesity (>97th percentile)	4.9%
Screw removal (%)	430 (85.5%)
Age at removal (years)	13.8 ± 1.2 (10–17)
Implant duration (years)	2.3 ± 0.6 (0.3–6)
Accessory procedures	39 (7.9%; 95% CI: 5.7–10.6%)
Complications	27 (5.5%; 95% CI: 3.5–7.5%)

**Table 2:** Patient-reported outcome measures at latest follow-up, including Foot and Ankle Ability Measure (FAAM) total score, percentage of ceiling effect, FAAM Activities of Daily Living (ADL), FADI pain subscale, and FAAM Sport subscale.

Outcome Variables	Value
FAAM total	98.8 ± 3.7 (75–100)
% ceiling effect	87.5% (95% CI: 71.2–82.4)
FAAM ADL	99.3 ± 3.2 (75–100)
FAAM Sport	98.0 ± 6.4 (75–100)

**Table 3:** Comparison of functional outcomes [including Foot and Ankle Ability Measure (FAAM) total score, percentage of ceiling effect, FAAM Activities of Daily Living (ADL), FADI pain subscale, and FAAM Sport subscale] and complication rates between patients who underwent screw removal and those with the screw retained at long-term follow-up. Values are reported as mean ± standard deviation or number of cases (%). *p*-values were calculated using the Mann–Whitney U test for ordinal variables and chi-square tests for categorical variables.

Variable	Screw Removal	Screw Retained	*p*-Value
Number of patients	430	64	
FAAM total	98.7 + 4.0	99.6 + 0.9	0.79
FAAM ADL	99.2 + 3.5	99.8 + 0.5	0.71
FAAM sport	97.6 + 6.9	99.7 + 0.9	0.47
TAS	3.1 + 1.6	3.8 + 2.1	0.08
Complications	22 (5.1%)	5 (7.8%)	0.38

## Data Availability

Anonymized clinical and radiographic data that support the findings of this study are available from the corresponding author upon reasonable request.

## Supplementary Materials

The following supporting information can be downloaded at https://www.mdpi.com/article/10.3390/children12081047/s1. Table S1: Differences in demographics and surgical details between responders and non-responders. Table S2: Differences in postoperative outcomes between males and females. Table S3: Differences in postoperative outcomes between patients with and without reported complications.

## Abbreviations

The following abbreviations are used in this manuscript:

**Abbreviation**	**Definition**
FFF	Flexible Flatfoot
SA	Subtalar Arthroereisis
C-Stop	Calcaneo-Stop
FAAM	Foot and Ankle Ability Measure
FAAM-ADL	FAAM—Activities of Daily Living Subscale
FAAM-Sport	FAAM—Sport Subscale
FADI	Foot and Ankle Disability Index
TAS	Tegner Activity Scale
BMI	Body Mass Index
CI	Confidence Interval
ICC	Intraclass Correlation Coefficient
MDC	Minimal Detectable Change
MCID	Minimal Clinically Important Difference
M/F	Male/Female
G*Power	G*Power (Statistical Power Analysis Software)
