# Supplementary material for: Ten-Year Clinical and Functional Outcomes of Anterograde Calcaneo-Stop Arthroereisis for Idiopathic Flexible Flatfoot in Children: A Single-Center Cohort Study
Source: Children (Basel). 2025 Aug 9;12(8):1047. doi: 10.3390/children12081047 (PMC12385076; doi:10.3390/children12081047)
Supplement: Supplementary file 1 [file children-12-01047-s001.zip › children-3761103-supplementary.pdf]

Table S1. Differences in demographics and surgical details between responders and non-responders. Statistically significant differences are highlighted in bold. [1]:Chi-square goodness-of-fit test for normality. [2]:Chi-square test for 2x2 contingency tables. [3]: Student's t-test with Levene's correction for normal distribution.

Variable	Responders (%)	Non-responders (%)	p-value
Patients	232 (47%)	262 (53%)	0.18[1]
Females	94 (40.5%)	92 (35.1%)	0.22[2]
Age (years)	11.6 ± 1.1	11.5 ± 1.1	0.36[3]
Weight (Kg)	45.5 ± 7.2	45.7 ± 8.0	0.78[3]
Height (cm)	151 ± 6.5	151 ± 7.2	0.67[3]
BMI	19.9 ± 2.2	19.9 ± 2.5	0.94[3]
Accessory procedures	15 (6.5%)	24 (9.2%)	0.32[2]
Screw removal	33 (14.2%)	31 (11.8%)	0.50[2]
Age at screw removal	13.8 ± 1.1	13.8 ± 1.2	0.99[2]
Compications	19 (8.2%)	8 (3.1%)	**0.02**[2]

Table S2. Differences in postoperative outcomes between males and females. Statistically significant differences are shown in bold. [1]: Chi-square goodness-of-fit test for normality. [2]: Mann–Whitney U test for comparison of ordinal variables.

Variable	Males	Females	Mean difference (95% CI)	p-value
Patients	138 (59.5%)	94 (40.5%)		**<0.01**[1]
FAAM total	99.5 ± 1.7	97.8 ± 5.3	1.6 ± 0.6 (0.5 – 2.7)	**<0.01**[2]
FAAM ADL	99.9 ± 0.5	98.3 ± 4.9	1.5 ± 0.5 (0.5 – 2-5)	**<0.01**[2]
FAAM Sport	98.8 ± 5.0	96.8 ± 8.0	2.0 ± 0.9 (0.2 – 3.8)	**<0.01**[2]
TAS	4.1 ± 2.2	3.1 ± 1.6	1.0 ± 0.2 (0.5 -1.5)	**<0.01**[2]

Table S3. Differences in postoperative outcomes between patients with and without reported complications. Statistically significant differences are shown in bold. [1]: Chi-square goodness-of-fit test for normality. [2]: Mann–Whitney U test for comparison of ordinal variables.

Variable	No Complications	Complications	Mean difference (95% CI)	p-value
Patients	213 (91.8%)	19 (8.2%)		**<0.01**[1]
FAAM total	99.0 ± 3.3	96.3 ± 6.5	2.7 ± 1.5 (-0.5 – 5.8)	**<0.01**[2]
FAAM ADL	99.4 ± 2.9	97.8 ± 5.8	1.6 ± 1.3 (-1.2 – 4.3)	**<0.01**[2]
FAAM Sport	98.4 ± 5.8	93.6 ± 10.5	4.8 ± 2.4 (-0.3 – 9.9)	**<0.01**[2]
TAS	3.7 ± 2.0	4.2 ± 2.0	-0.5 ± 0.5 (-1.5 – 0.4)	0.23[2]
